# Topological embedding and directional feature importance in ensemble classifiers for multi-class classification

**DOI:** 10.1016/j.csbj.2024.11.013

**Published:** 2024-11-13

**Authors:** Eloisa Rocha Liedl, Shabeer Mohamed Yassin, Melpomeni Kasapi, Joram M. Posma

**Affiliations:** a Section of Bioinformatics, Department of Metabolism, Digestion and Reproduction, Faculty of Medicine, Hammersmith Hospital Campus, Imperial College London, London, W12 0NN, United Kingdom; b Department of Surgery and Cancer, Faculty of Medicine, Hammersmith Hospital Campus, Imperial College London, London, W12 0NN, United Kingdom; c Centre for Integrative Systems Biology and Bioinformatics (CISBIO), Department of Life Sciences, Faculty of Natural Sciences, South Kensington Campus, Imperial College London, London, SW7 2AZ, United Kingdom; d Section of Nutrition, Department of Metabolism, Digestion and Reproduction, Faculty of Medicine, Hammersmith Hospital Campus, Imperial College London, London, W12 0NN, United Kingdom

**Keywords:** Decision trees, Feature importance, Machine learning, Multi-class classification, Topological information

## Abstract

Cancer is the second leading cause of disease-related death worldwide, and machine learning-based identification of novel biomarkers is crucial for improving early detection and treatment of various cancers. A key challenge in applying machine learning to high-dimensional data is deriving important features in an interpretable manner to provide meaningful insights into the underlying biological mechanisms

We developed a class-based directional feature importance (CLIFI) metric for decision tree methods and demonstrated its use for The Cancer Genome Atlas proteomics data. The CLIFI metric was incorporated into four algorithms, Random Forest (RF), LAtent VAriable Stochastic Ensemble of Trees (LAVASET), and Gradient Boosted Decision Trees (GBDTs), and a new extension incorporating the LAVA step into GBDTs (LAVABOOST). Both LAVA methods incorporate topological information from protein interactions into the decision function.

The different models' performance in classifying 28 cancers resulted in F1-scores of 92.6% (RF), 92.0% (LAVASET), 89.3% (LAVABOOST) and 85.7% (GBDT), with no method outperforming all others for individual cancer type prediction. The CLIFI metric enables visualisation of the model's decision-making functions. The resulting CLIFI value distributions indicated heterogeneity in the expression of several proteins (MYH11, ER*α*, BCL2) across different cancer types (including brain glioma, breast, kidney, thyroid and prostate cancer) aligning with the original raw expression data.

In conclusion, we have developed an integrated, directional feature importance metric for multi-class decision tree-based classification models that facilitates interpretable feature importance assessment. The CLIFI metric can be combined with incorporating topological information into the decision functions of models to introduce inductive bias, enhancing interpretability.

## Highlights

• Development of a directional feature importance metric for decision tree models.
• Metric integrated in multi-class ensemble classification models for improved feature importance assessment.
• Incorporating inductive bias into decision functions with topological information improves model interpretability.

## Graphical abstract

**Figure:**
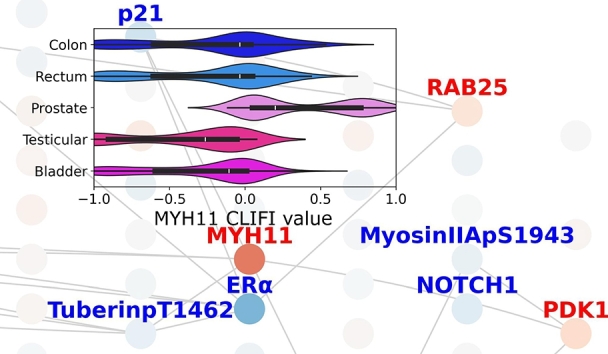

## 1 Introduction

Cancer has risen from third place in 2010 to second place in 2019 as the leading cause of disease-related death worldwide, and it is forecast that the global cancer burden will continue to grow for the next two decades [1], [2], [3]. As a result, the application of machine learning (ML) methods in oncology has expanded [4]. In addition to data analysis automation, ML models often show higher accuracy in diagnosis and survival predictions than traditional clinical methodology [5], [6]. Given the shift in cancer medical practice to personalised and targeted treatments, classification of cancer patients is moving towards the need of identifying subpopulations within one cancer type [7], [8].

Ensemble methods, a subfield of ML, are commonly used for this classification task [9], [10], [11], as they excel in maintaining performance with high-dimensional datasets that have a small number of samples relative to the number of features (small *n*, large *p*). In contrast, other ML methods, such as deep learning algorithms require more samples for effective training [12]. Such high-dimensional datasets are common in cancer research, including genomic, epigenetic, transcriptomic, proteomic, and clinical data. Examples of ensemble methods include Random Forests (RFs) [13] and Gradient Boosted Decision Trees (GBDTs) [14]. Despite gradient boosting showing superior prediction performance in previous cancer prediction studies compared to RF, support vector machines, and logistic regression algorithms [15], [16], [17], the existence of cancer subtypes limits the application of RF and GBDT in cancer. While binary classification models can still be easily interpreted in how predictions are being made, by using Gini values, multi-class classification algorithms are not [18].

High-dimensional datasets often contain correlated features (feature interactions), which can lead the model to overfitting, by capturing noise or redundant features. Hence, they compromise model generalisation by assigning importance to non-biologically meaningful features [18]. There are techniques that can address the problem of correlated features e.g. Boruta algorithm [19], a novel RF algorithm, but they do not explicitly incorporate domain knowledge of correlations. For example, the Boruta algorithm was utilised to identify microRNAs distinguishing normal and ovarian cancer patients, yielding more differentially expressed microRNAs compared to previous studies [20]. Subsequent pathway analysis validated the ML findings, demonstrating concordance with existing literature [20]. While these algorithms are effective, biological pathway interpretation and validation are necessary to verify and interpret the results. Topological ML algorithms incorporating feature interaction information (such as microRNA interactions) into the model's architecture can help solve the multicollinearity problem, thereby enhancing the model's interpretability and reducing the need for extensive post hoc validation analyses.

Informative feature importance values depend on two factors: class-specificity and directionality. ML models in cancer research commonly utilise binary classification models with Gini-based feature importance assignment, which considers only the magnitude of features' influence on predictions [10], [21], [22]. Applying multi-class classification algorithms offer greater versatility as they enable prediction of multiple classes using a single model.

In cancer research, ensemble methods with RF and GBDTs are rarely used for multi-class classification, instead a binary one versus all approach is usually employed to identify class-specific important features. For example, Ortiz-Ramon et al. used two RF models to discriminate brain metastases patients, based on their primary site of origin: a multiclass RF model with 87% accuracy and a one versus all RF model with 82% accuracy [23]. They then, identified class-specific Gini-based feature importance by analysing the one vs all models. While the Gini-based feature importance did help identify differentiating biomarkers, it did not clarify whether high or low values of the biomarkers are associated with each class. The need for class-specific and directional feature importance assignment is essential for broader application of multi-class ensemble models. Directional feature importance calculations are available for ensemble models, for example permutation importance [13] and SHapley Additive exPlanations (SHAP) [24]; however, they are post-hoc and not incorporated within the methods. To the best of our knowledge, no such method exists for RF or GBDT that is integrated within algorithm.

Our contribution aims to address two gaps. The first is to introduce an integrated, directional feature importance metric for decision tree-based models (such as RF and GBDT) to facilitate feature importance assessment for multi-class classification. We demonstrate the feature importance measure on the small-scale Fisher Iris data before applying it on a large dataset. The second is to expand on recently published work, LAVASET (LAtent VAriable Stochastic Ensemble of Trees) [25], that demonstrated how data-specific properties can be integrated in the ensemble model for better interpretability for correlated, temporal and spatial features. We extend this paradigm to GBDTs, producing LAVABOOST (LAtent VAriable gradient BOOSTed decision trees), and apply the 4 algorithms for multi-class classification in The Cancer Genome Atlas (TCGA) proteomics dataset [26] of 28 different cancers with incorporation of topological information of protein-protein interactions.

## 2 Materials and methods

### 2.1 Data

#### 2.1.1 Data - Fisher Iris

The original Fisher Iris data was obtained from scikit-learn and used to demonstrate the directional feature importance. Here, we added 3 random noise features of different distributions (Gaussian, uniform, and bimodal) to the original features (sepal length, sepal width, petal length, petal width) to test the feature importance for features with different conditions of (random) distributions (we refer to this dataset as ‘Iris’). Additionally, we randomly shuffled the values for each feature to break the correlation pattern of the Iris data to demonstrate the directional feature importance output for models without predictive power, this dataset is referred to as ‘Iris-permuted’.

#### 2.1.2 Data - TCGA

Proteomic profiling data of 28 cancer types was obtained from TCGA project. These publicly available datasets were generated from tumour tissue analysis conducted by the University of Texas MD Anderson Cancer Centre using reversed-phase protein arrays [26]. Table 1 outlines the total number of samples per cancer included in the initial dataset. All samples were obtained from the TCGA portal (version 39.0, release date 04/Dec/2023) at https://portal.gdc.cancer.gov/.

**Table 1:** Data set description.

Cancer type	Abbreviation	Number of samples
glioblastoma multiforme	GBM	243
brain lower grade glioma	LGG	435
head and neck squamous cell carcinoma	HNSC	354
thyroid carcinoma	THCA	381
oesophageal carcinoma	ESCA	126
sarcoma	SARC	226
skin cutaneous melanoma	SKCM	352
lung adenocarcinoma	LUAD	365
lung squamous cell carcinoma	LUSC	328
mesothelioma	MESO	62
thymoma	THYM	90
breast invasive carcinoma	BRCA	919
stomach adenocarcinoma	STAD	357
liver hepatocellular carcinoma	LIHC	184
pancreatic adenocarcinoma	PAAD	120
adrenocortical carcinoma	ACC	46
pheochromocytoma and paraganglioma	PCPG	82
kidney renal clear cell carcinoma	KIRC	478
kidney chromophobe	KICH	63
kidney renal papillary cell carcinoma	KIRP	216
colon adenocarcinoma	COAD	363
rectum adenocarcinoma	READ	132
ovarian serous cystadenocarcinoma	OVCA	432
uterine corpus endometrial carcinoma	UCEC	440
cervical squamous cell carcinoma and endocervical adenocarcinoma	CESC	172
prostate adenocarcinoma	PRAD	352
testicular germ cell tumours	TGCT	122
bladder urothelial carcinoma	BLCA	343

#### 2.1.3 Data processing - TCGA

Proteins with more than 50% missing values were removed. Missing values were imputed using the k-Nearest Neighbours Imputer (scikit-learn, v1.3.2) with k=5 (default value - different values were assessed (3, 5, 7) but from the unsupervised analysis these did not seem to result in any observed differences in the projections). The protein list was further refined to include only those that had been validated by Western blotting in a TCGA pan-cancer independent study [27]. Compared to the prior study, we included additional cancer types. To assess whether this affected clustering ability, an unsupervised analysis of the data with and without the additional cancer types showed no clear differences. Consequently, the final dataset comprised of 7,783 samples with 113 proteomic features.

### 2.2 Machine learning classifiers

#### 2.2.1 Random forest and LAVASET

The LAVASET package provides implementation for both the RF and LAVASET algorithms [25], based on the principles of Breiman's original RF algorithm [13]. LAVASET utilises the original C++ implementation for computational efficiency, with main functions written in Python for easier user readability. The traditional Classification And Regression Trees (CART) algorithm [28] is used to build individual decision trees. The modifiable hyperparameters for both algorithms are the number of trees, number of samples per tree, and the maximum number of features. In addition, LAVASET has a distance parameter which defines the threshold strength of feature interactions to be considered in the embedding.

The ‘LAVA’ step occurs before the feature is chosen for the best split (see [25] for complete details). In brief, Principal Component Analysis (PCA) is used to identify the first right singular vector (loading) of the decomposition of the feature selected and its correlated features. The loading is then used to transform the original features into a single score (left singular value). This is performed for all features (and their neighbours/correlated features) selected at the splitting step. Therefore, the feature dataset is transformed to consist of latent variables instead of the original feature values.

#### 2.2.2 Extension of latent variable embedding to boosting algorithms

The multiclass classification gradient boosting decision tree (GBDT) algorithm developed in this study was adapted from Matt Bowers' implementation (https://github.com/mcb00/rr-blog/tree/main/posts/gradient-boosting-machine-with-any-loss-function) of Algorithm 6 from the greedy function approximation [14]. This implementation uses a one-against-all approach to reduce the problem into K binary problems, where K is the number of classes. Our implementation of GBDT, as well as LAVABOOST (Latent Variable embedding into GBDTs), uses the traditional CART algorithm [28] with the original C++ implementation to build individual decision trees rather than the scikit-learn DecisionTreeRegressor function. The sequential trees in GBDTs and LAVABOOST predict the errors of the previous prediction, therefore, to prevent biases, the predictions are initialised to 0.

LAVABOOST has a number of prerequisites and hyperparameters that can be optimised (see section 2.4). A distance matrix is provided by the user containing the strength of interactions between features in the input data set (see section 2.2.3). The features the algorithm considers correlated are those with an interaction strength less than or equal to the threshold set by the user, the threshold is set by the distance parameter. The modifiable optimisation parameters include the number of estimators (boosting rounds), learning rate, number of samples per tree, and the maximum number of features. When the number of samples per tree or maximum number of features is set to None, all samples/features, respectively, are used.

#### 2.2.3 Distance metric for latent variable embedding

We produced a distance matrix representing the protein interactions to use as input to the distance parameter of LAVASET and LAVABOOST. The Search Tool for the Retrieval of Interacting Genes/Proteins database (STRINGdb) version 12 (https://string-db.org/) [29] was used to identify the protein-protein interactions of all proteins in the TCGA dataset. For any TCGA protein name not found in the STRINGdb database, we used the gene name of each protein (listed in [27]) to find protein interactions between all 113 features. The distance matrix was initialised by including all protein self-interactions, proteins linked to the same gene, and protein-coding gene-gene interactions (evaluated using confidence level of 0.7 in STRINGdb) as adjacencies. The distance parameter was set to 1 to ensure that for each protein ‘j’, the set of features used in the ‘LAVA’ step were those proteins that were linked the gene of protein j and/or those proteins whose gene has a gene-gene interaction with the gene from protein j only.

### 2.3 Directional feature importance

The directional feature importance calculation occurs after the optimal feature (or latent feature for LAVASET and LAVABOOST) has been selected with the optimal split. For all splits, the Gini coefficient values are calculated. For RF and GBDT, the Gini is attributed to the chosen feature for the optimal split. For LAVASET and LAVABOOST, a normalised Gini feature importance is calculated for each original feature using the absolute value of the loadings (normalised to a sum of 1). Below we describe the newly proposed CLass-based Integrated directional Feature Importance (CLIFI) calculation for ensemble models such as RF, GBDT, LAVASET and its novel extension LAVABOOST. The benefit of this calculation is that it is integrated into the algorithm and therefore feature importances of the models can be directly inferred without need for additional (post-hoc) techniques.

The G-test (Eqn. (1)), a complimentary approach to Kullback-Leibler divergence, is used to compare the observed and expected frequencies of categorical data (i.e. the distribution of groups after splitting). Where $O_{i}$ is the observed count for the i^th^ category and likewise, $E_{i}$ is the expected count for the same category under the null hypothesis.

(1) $$G=2\times \sum_{i=1,\forall O_{i}>0}^{n}O_{i}\times ln\frac{O_{i}}{E_{i}}$$

We use a modified version of the G-test to quantify the distribution of samples in a class in each of the splits, where the observed count in Equation (1) is replaced with the frequency in the left split ($L_{i}$) for class i (Eqn. (2)), and the expected count is replaced by half the frequency in the parent node ($\frac{1}{2}P_{i}$). The same process is done for frequency in the right split ($R_{i}$, Eqn. (3)). These two quantities are partial G-tests ($M_{ij}$), and summing these values results in the combined G-test value for that class. This assumes that $P_{i}$, and either (or both) of $L_{i}$ and $R_{i}$, are non-zero.

(2) $$M_{i1}=2\times L_{i}\times ln\frac{2\times L_{i}}{P_{i}}$$

(3) $$M_{i2}=2\times R_{i}\times ln\frac{2\times R_{i}}{P_{i}}$$

A perfect split is represented by $2\times P_{i}\times ln2$, therefore we normalise *M* by the perfect split to scale it between 0 (equal split) and 1 (perfect split) and subsequently multiply with $P_{i}/A_{i}$ to account for the number of samples of the class considered for the split relative to all samples in that tree ($A_{i}$, ancestor node). The directionality of the feature for class *i* is indicated by the sign of $M_{i2}-M_{i1}$. Combining this, and simplifying, results in Equation (4) for the CLIFI of feature *j* for class *i*:

(4) $$CLIFI_{ij}=\frac{R_{i}\times ln\frac{2\times R_{i}}{P_{i}}+L_{i}\times ln\frac{2\times L_{i}}{P_{i}}}{A_{i}\times ln2}\times sign\left(R_{i}\times ln\frac{2\times R_{i}}{P_{i}}-L_{i}\times ln\frac{2\times L_{i}}{P_{i}}\right)$$

A positive CLIFI value for a feature signifies that the direction of the split is weighted more towards the right node (higher split values) than the left (lower split values). The magnitude of the association is given by the numerical value itself, where the closer is it to 1 (or -1), the more it is associated with higher (or lower) values of that feature for RF and LAVASET models. The aggregated CLIFI (aCLIFI) value is the sum of the CLIFI values across all trees for a specific feature and class, and for comparison between models the normalised aggregated CLIFI (naCLIFI) values represent a division by the highest absolute aggregated CLIFI value across all features and classes.

For GBDT and LAVABOOST the above cannot be used directly because each class label (*i*) predicted by the model is an error label, therefore, samples of the same class (noted by *h*) may have different error labels. Therefore, the CLIFI for these models is calculated by scaling the CLIFI value for the error label by the proportion of samples in the real class (Eqn. (5)), where *x* represents the distinct error labels associated with a class *h*, and *S* is the number of samples within a class *h* with error label *i* divided by the total number of samples in class *h*.

(5) $$CLIFI_{hj}=\sum_{\forall i\in x}S_{hi}\times CLIFI_{ij}$$

### 2.4 Model evaluation

30% of the data was set aside for testing, with the remaining 70% split 80:20 into training and validation sets. Individual class frequencies were balanced across the subsets of data. The training set (ca. 56% of the total data) was used to calculate individual models with different hyperparameter settings, and the validation set (ca. 14% of the total data) was used to determine the optimal hyperparameters. The optimal model was then used to predict the left-out test set (30%). For the TCGA data, RF, LAVASET, and GBDT were run 100 distinct times and LAVABOOST 10 distinct times with different random states to evaluate the model robustness.

The optimal hyperparameters for RF and LAVASET models were models with 150 trees and sqrt (square root) of number of features for each split. The values trialed for number of trees were 100 to 180 in steps of 10. Both models used 80% of samples per tree. Optimal parameters for GBDT and LAVABOOST were 130 estimators (boosting rounds), a learning rate of 0.1, sqrt of number of features for each split, and including all samples (None). The values trialed for number of trees were 10 to 150 in steps of 10, and the values trialed for learning rate were 0.1, 0.2, and 0.3. The distance parameter for LAVABOOST and LAVASET was set to 2.

Classification performance was assessed based on accuracy, precision, recall and F1-score, with the mean ± standard deviation reported for each model, for the test set. The proximity matrix was used as input to UMAP (number of components=2, init=random, random state=0) [30] to visualise the similarity between samples from different cancer types.

The Iris dataset was split 80:20 into training and validation sets. The optimal parameters for the Iris data were the sqrt of number of features per split for RF and GBDT, 100 trees for RF, and 7 boosting rounds with 0.1 learning rate for GBDT.

### 2.5 Evaluation and visualisation of CLIFI values

Class feature importance assignment was evaluated for LAVASET, RF, LAVABOOST, and GBDT using the python package NetworkX. A template protein layout on a hexagonal grid was defined based on the protein interactions, with interacting proteins being closer together, and was used for all algorithms. The feature importance results present the top 10 proteins with the highest CLIFI value (and higher than the average CLIFI value of the positively assigned proteins). Negatively assigned proteins are presented in a similar manner (10 lowest CLIFI values that are lower than the average CLIFI of negative CLIFI values), with LAVASET and LAVABOOST also showing protein interactions between these proteins. For visualisation purposes that was restricted to the top 10 to allow all edges to be drawn without node-edge crossings.

Paired and unpaired t-tests were used to assess differences between methods in model performance. A Kruskal-Wallis test was used to evaluate whether features' CLIFI values were significantly different between classes. For those features with significant differences, a Mann-Whitney U test (Wilcoxon rank sum test) was used for pairwise comparisons and these were visualised in a heatmap. All p-values were adjusted for multiple testing using the Benjamini-Hochberg method.

### 2.6 Compute, software versioning, and code availability

All calculations were performed on an HP Z6 G4 workstation with 16-core Intel(R) Xeon(R) Silver 4110 CPU @ 2.10 GHz with 128 GB RAM.

Combining the TCGA study datasets and defining the protein interactions were implemented with R in (v4.3.2) R Studio (v2023.09.1+494). These codes are available from https://github.com/EloisaRL/TCGA-proteomics-pipeline.

All other analyses were implemented in Python (v3.12.1) with libraries and packages: lavaset (v0.1.1), matplotlib (v3.8.2), networkx (v3.2.1), numpy (v1.26.2), pandas (v2.1.4), plotly (v5.19.0), scikit-learn (v1.3.2), seaborn (v0.13.2), umap (v0.1.1), and umap-learn (v0.5.5).

The code for GBDT and LAVABOOST, and implementation of the direction feature importance for all 4 algorithms (including RF and LAVASET), have been added as separate branch to the LAVASET repository (https://github.com/melkasapi/LAVASET, v1.0.0).

## 3 Results

### 3.1 Classification performance

#### 3.1.1 Iris data

We show that our algorithms show consistent performance with respect to the example datasets, where the permuted version has low classification rates, and for the real data we show that RF outperforms GBDT (p < 0.0001) (Table 2). For the demonstration of the CLIFI values, we choose to show these for the RF models for both the Iris (predictive) and Iris-permuted (not predictive) datasets.

**Table 2:** Classification performance of RF and GBDT for the Iris and Iris-permuted datasets. Scores for accuracy, precision (weighted), recall (weighted), F1-score (weighted) are given across 20 random initialisations of the models. The RF was run with 100 trees, GBDT with 7 boosting rounds and learning rate of 0.1. Values shown are the mean ± standard deviation, represented as percentages. Values in bold indicate the highest performances for each metric.

Dataset	Algorithm	Accuracy (%)	Precision (%)	Recall (%)	Macro F1 (%)
Iris	RF	**90.00** ± 0.00	**92.73** ± 0.00	**90.00** ± 0.00	**91.34** ± 0.00
Iris-permuted	RF	38.50 ± 2.68	50.65 ± 7.00	38.50 ± 2.68	43.68 ± 4.25
Iris	GBDT	72.33 ± 1.53	72.39 ± 1.12	72.33 ± 1.53	72.36 ± 1.27
Iris-permuted	GBDT	29.00 ± 2.13	26.80 ± 4.23	29.00 ± 2.13	27.77 ± 3.26

#### 3.1.2 TCGA data

For the TCGA dataset (28 classes), RF outperformed the other methods (Table 3) though while its difference with LAVASET is significant (p < 0.001) the difference is small. Both RF and LAVASET outperform GBDT and LAVABOOST, with LAVABOOST outperforming GBDTs (p < 0.001). LAVABOOST appears to have larger variability in its performance compared to LAVASET. The class-specific performance varies between 0% to 100% for all algorithms (see Appendix A), with no model consistently outperforming other models on all cancer types. For example, while RF and LAVASET have a considerable higher overall accuracy, for glioblastoma, brain lower grade glioma, thyroid, and testicular germ cell cancer the boosting methods have better performance.

**Table 3:** Classification performance of RF, LAVASET, GBDT, and LAVABOOST. Scores for accuracy, precision (weighted), recall (weighted), F1-score (weighted) across 100 random initialisations of each model. Values shown are the mean ± standard deviation. Values in bold indicate the highest performances for each metric.

Algorithm	Accuracy (%)	Precision (%)	Recall (%)	Macro F1 (%)	Time/model (min)
RF	**93.07** ± 0.11	**92.20** ± 0.35	**93.07** ± 0.11	**92.63** ± 0.22	3.15
LAVASET	92.35 ± 0.14	91.76 ± 0.39	92.35 ± 0.14	92.05 ± 0.26	**2.59**
GBDT	86.52 ± 0.22	86.33 ± 0.26	86.52 ± 0.22	85.65 ± 0.26	33.58
LAVABOOST	89.26 ± 0.21	88.96 ± 0.59	89.26 ± 0.21	89.11 ± 0.38	254.04

### 3.2 Feature importance assessment

#### 3.2.1 Iris data

**Fig. 1:**
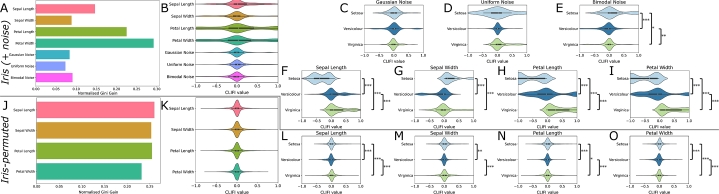
Visualisation of feature importance for Iris (A-I) and Iris-permuted (J-O) datasets for the RF model. (A) Normalised Gini coefficients for the Iris dataset. (B) Aggregated distribution of CLIFI values across all classes, CLIFI distributions that deviate from a normal distribution around 0 may have class information. (C-E) Distribution of CLIFI values for Gaussian (C), uniform (D) and bimodal (E) noise for the Iris dataset. (F-I) Distribution of CLIFI values for sepal length (F), sepal width (G), petal length (H), and petal width (I) for the Iris dataset. (J) Normalised Gini coefficients for the Iris-permuted dataset. (K) Aggregated distribution of CLIFI values across all classes shows no deviations from 0. (L-O) Distribution of CLIFI values for sepal length (L), sepal width (M), petal length (N), and petal width (O) for the Iris-permuted dataset. Pairwise comparisons within a class were tested. *** = p < 0.0005, ** = p < 0.005, * = p < 0.05.

The CLIFI value importance assignment was first tested on the Iris dataset to serve as a benchmark for demonstrating the interpretability. Fig. 1A shows the output from using the Gini coefficient, indicating that sepal length (SL), petal length (PL), and petal width (PW) are most predictive. In the CLIFI distribution plot (Fig. 1B) PL, PW and SL have the broadest spread of CLIFI values, i.e. they deviate most from 0, hence these are likely to be predictive in the model. Fig. 1C-E shows the distribution of CLIFI values for the 3 noise variables for each class (Setosa, Versicolour, Virginica) is centred at 0, and while come noise variables had higher Gini coefficients than the real sepal width (SW) feature there are no significant differences between groups in the CLIFIs. The largest differences are seen for the real variables (Fig. 1F-I). These distributions clearly show the relationship between individual features and the class information.

**Fig. C.9:**
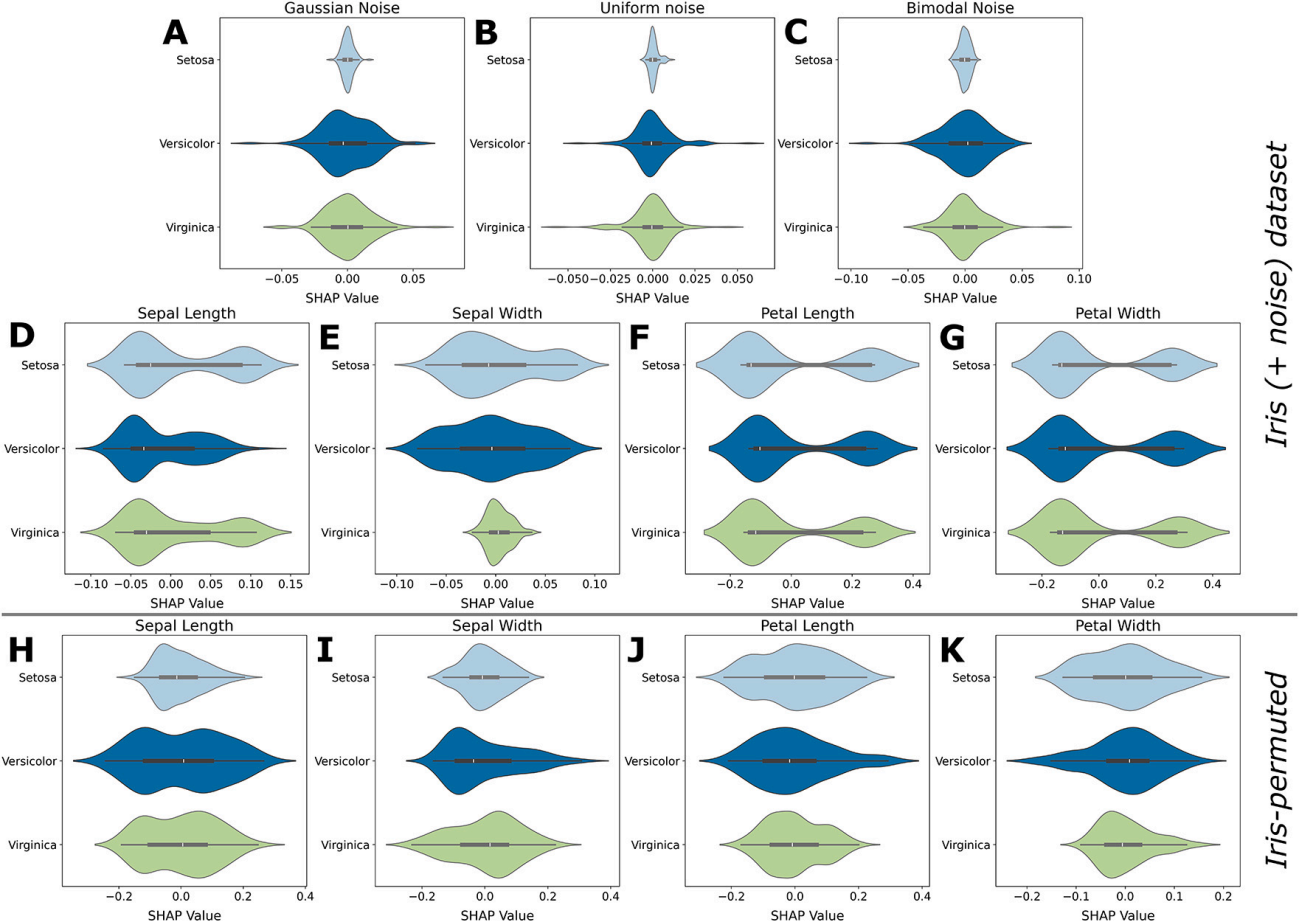
Visualisation of SHAP feature importance for Iris and Iris-permuted dataset for the RF model to accompany Fig. 1. (A-G) Iris data. (H-K) Iris-permuted dataset. (A-C) Distribution of SHAP values for Gaussian (A), uniform (B), and bimodal (C) noise. (D-G) Distribution of SHAP values for sepal length (D), sepal width (E), petal length (F), and petal width (G) for the Iris dataset. (H-K) Distribution of SHAP values for sepal length (H), sepal width (I), petal length (J), and petal width (K) for the Iris-permuted dataset.

While the Gini coefficients are still non-zero (Fig. 1J) for Iris-permuted, the CLIFI values are all centred around 0 (Fig. 1K) indicating an equal split. The differences in the permuted features (Fig. 1L-M) are all centred around 0 and considerably narrower than those from the real model (Fig. 1F-I), hence features with a wider dispersion of CLIFI values away from zero (toward either -1 or 1) signify greater predictive importance in class separation. The comparison with SHAP values is given in Fig. C.9.

#### 3.2.2 TCGA data - feature importance visualisation

**Fig. 2:**
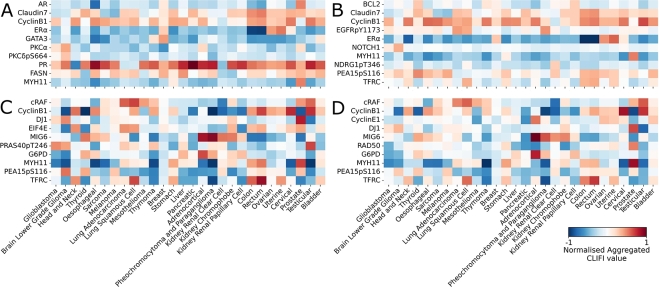
Heatmap of the top ten most important proteins across all 28 cancer types. Proteins selected based on the highest absolute normalised aggregated CLIFI value. (A) RF, (B) LAVASET, (C) GBDT, and (D) LAVABOOST. Colour bar shows the feature importance gradient, from negative to positive CLIFI-values, with white indicating a zero value.

Analysis of the topmost important proteins across all cancers shows that RF and LAVASET have the capability to detect proteins with similar average CLIFI magnitudes of importance across multiple cancer types, e.g. progesterone receptor (PR) in RF, and cyclin B1 and ER*α* (oestrogen receptor alpha) in both RF and LAVASET. We visualised the aggregated CLIFI values (normalised to the maximum value to allow comparison between methods) as heatmaps in Fig. 2.

Notably, within these proteins with high naCLIFI magnitude concordance, there are certain cancer types with significantly different naCLIFI values when compared to the majority. For example, Fig. 2A shows that while the average naCLIFI values for PR is close to 1 in most cancer types, in lung adenocarcinoma, lung squamous cell, and kidney renal clear cell, the average naCLIFI value is close to -1. Additionally, Fig. 2B shows that while the average naCLIFI value for Cyclin B1 is close to 1 in most cancer types, in kidney renal clear cell the average naCLIFI value is close to -1. However, this is not seen in the GBDT or LAVABOOST (Fig. 2C, D), instead most proteins have a high CLIFI value magnitude for some cancer types while the others have varying magnitudes. For example, the MYH11 protein has a high magnitude of naCLIFI values for thymoma (<0) and prostate cancer (> 0) in all models, with the boosting models showing greater variability due to these predicting errors of prior trees.

**Fig. B.7:**
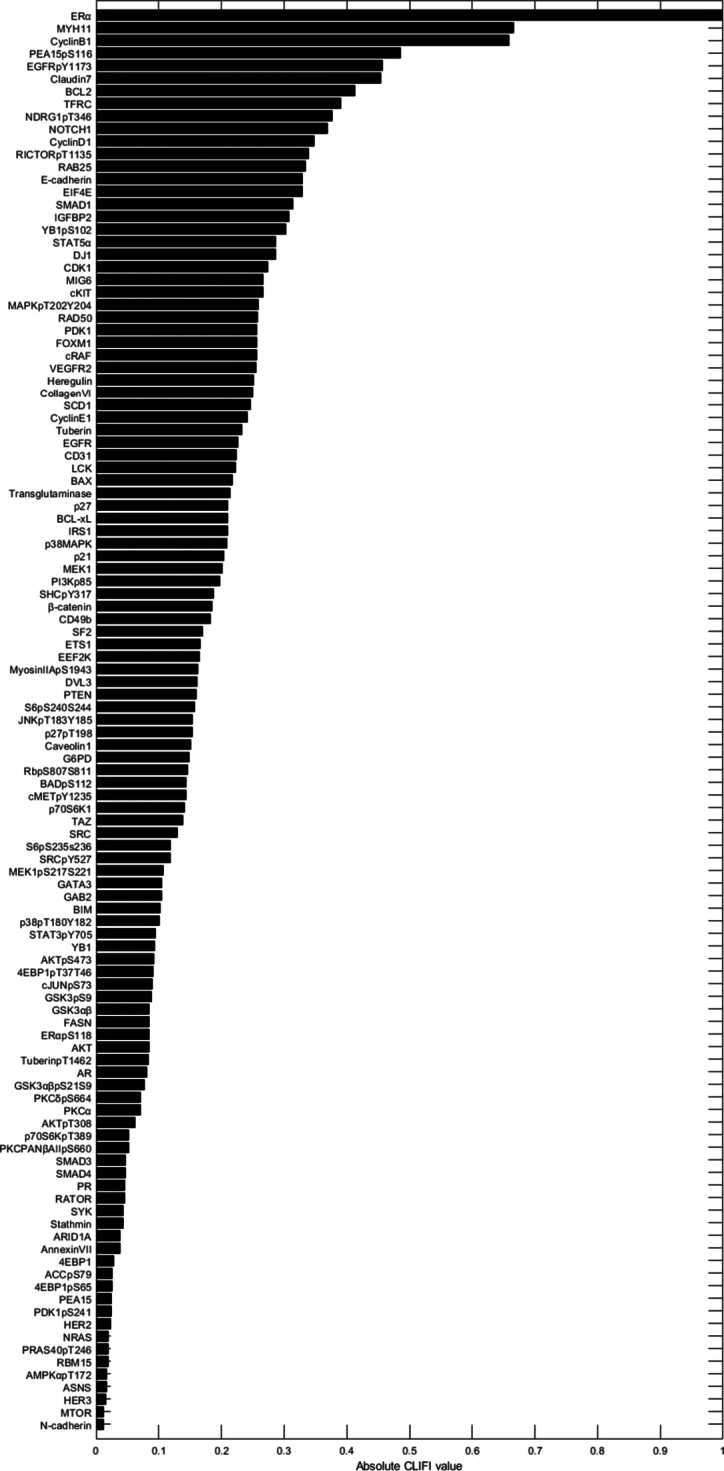
Bar plot of the absolute CLIFI values of the LAVASET model. The largest relative drops were chosen as cutoffs for the feature ablation presented in Table 4.

CLIFI allows for ranking features based on importance as with other feature importance metrics (Fig. B.7). We evaluated the relative drops in importance based on the absolute CLIFI values and selected 5 cutoffs for a feature ablation study with LAVASET. Dropping the bottom 14 and 25 features (least important) resulted in models with similar performance to the full model (Table 4). Dropping the top 10, 18 and 46 features did result in drops in performance indicating that CLIFI is able to identify relevant features.

**Table 4:** Ablation of features for LAVASET. Features were ranking based on absolute CLIFI value then the top or bottom n features were removed, and models were calculated using the same random states. Five cutoff points were selected based on bar plot (Fig. B.7) of descending CLIFI values using the elbow method. Scores for accuracy and F1-score (weighted) across 100 random initialisations of each model. Values shown are the mean ± standard deviation.

Features	Accuracy (%)	Precision (%)	Recall (%)	Macro F1 (%)
All	92.35 ± 0.14	91.76 ± 0.39	92.35 ± 0.14	92.05 ± 0.26
drop 100-113	92.06 ± 0.10	91.13 ± 0.34	92.06 ± 0.10	91.59 ± 0.20
drop 89-113	92.26 ± 0.13	92.52 ± 0.44	92.26 ± 0.13	92.39 ± 0.26
drop 1-10	91.51 ± 0.15	90.43 ± 0.21	91.51 ± 0.15	90.97 ± 0.16
drop 1-18	91.10 ± 0.19	90.00 ± 0.19	91.10 ± 0.19	90.55 ± 0.19
drop 1-46	88.56 ± 0.20	87.90 ± 0.39	88.56 ± 0.20	88.23 ± 0.27

#### 3.2.3 TCGA data - interpretability of feature importance in latent variable embedding models

**Fig. 3:**
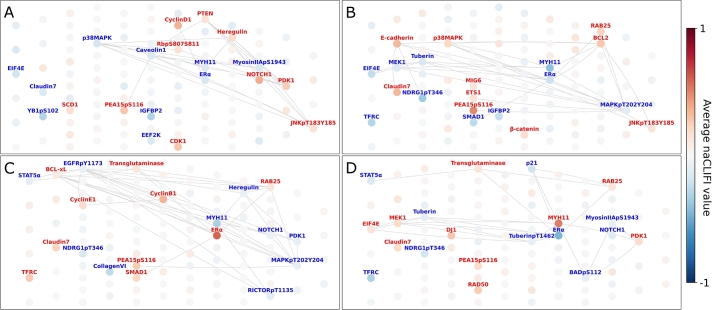
Visualisation of important features (node colour) and protein-protein interactions (edges) for selected cancer types from the LAVASET multi-class classification model. (A) Brain lower grade glioma (F1 = 0.97). (B) Thyroid cancer (F1 = 0.98). (C) Uterine endometrial cancer (F1 = 0.95). (D) Prostate adenocarcinoma (F1 = 1.00). Edges are only shown if labelled proteins have interactions.

While the heatmaps show the overall importance, these plots fail to display the relations between features. LAVASET and LAVABOOST integrate topological information, hence the outputs are conditional on the mapping of features. Fig. 3 shows the top proteins for four cancer types for which LAVASET outperformed all other methods. The edges show the neighbours in the distance matrix for calculating the latent embedding to facilitate interpreting the feature relations.

It can be seen that while MYH11 (smooth muscle myosin heavy chain 11) has interactions with BCL2 (B-cell lymphoma 2) and p38MAPK (p38 mitogen-activated protein kinase), and ER*α* with RAB25 (Ras-related protein in brain 25) and SMAD1 (Mothers Against Decapentaplegic Homolog 1), this is independent from the signs of the naCLIFI values. Incorporating the topological information allows for interpretation of the model in terms of (joint) function and role rather than on an individual protein basis (as with RF and GBDT). More relations between proteins are incorporated into the model, however here we only visualise the top few for simplicity.

#### 3.2.4 TCGA data - individual feature importance evaluation

**Fig. 4:**
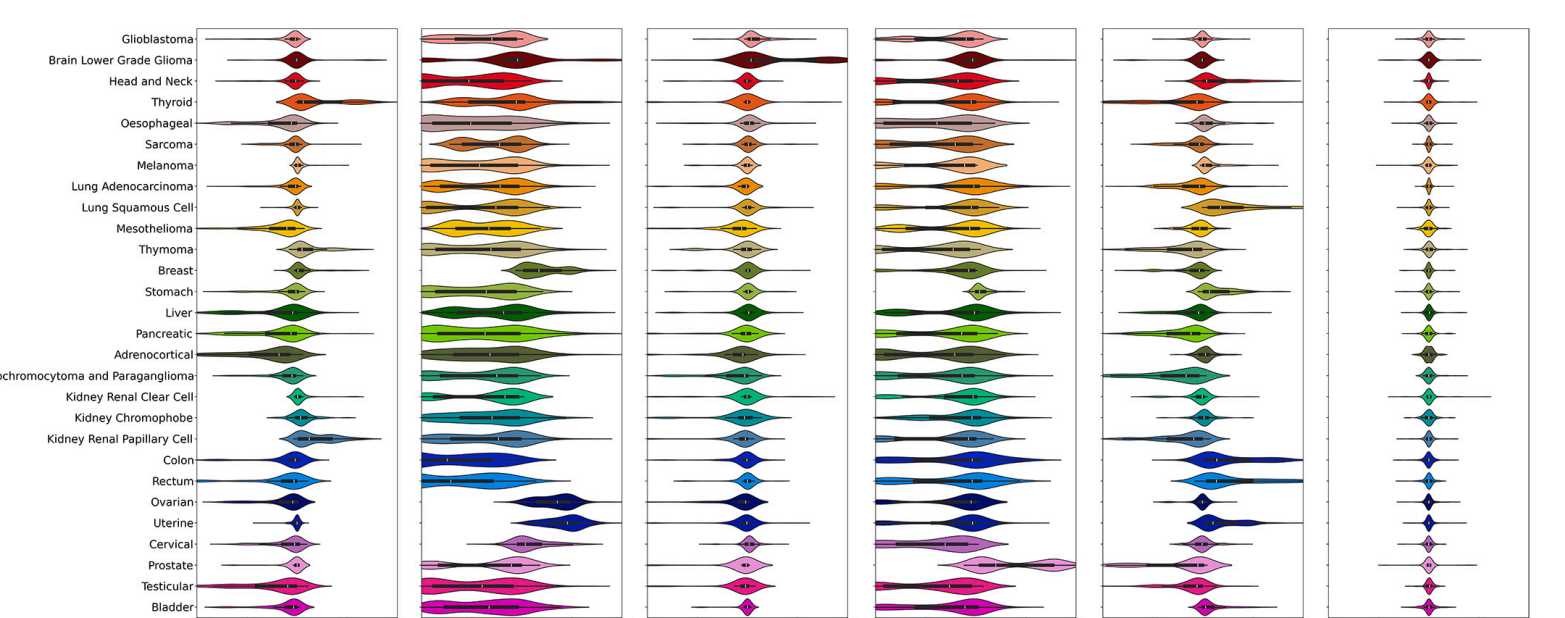
Visualisation of CLIFI distributions with violin plots of five selected top proteins, and one unimportant, from the LAVASET multi-class classification model. From left to right: BCL2, ER*α*, NOTCH1, MYH11, TFRC, MTOR. Violin plots of SHAP values for the same proteins can be found in Fig. C.10.

Similar to the Iris data, the CLIFI values allow for the visualisation of feature importance across all classes for individual features, with the distribution indicating the sign of association with the split (Fig. 4). As example, we show several proteins with different distributions of CLIFI values.

For example, the distribution of CLIFI values for BCL2 indicates that it is importance in the classification of several cancers, each of which appear to have bi- or multimodal distributions (hinting at heterogeneity), and specifically with thyroid and kidney renal papillary cell (higher expression) and oesophageal, mesothelioma, liver, pancreatic and adrenocortical (lower expression) cancers. TFRC (transferrin receptor) shows CLIFI distributions trending in opposite directions for lung squamous cell carcinoma (higher) and lung adenocarcinoma (lower) indicating potentially discriminatory potential between different cancers of the same organ.

Other proteins such as ER*α* and MYH11 have clear patterns for specific cancers with higher and lower expression of these proteins, with the bimodal distributions more evident that for other proteins. NOTCH1 (neurogenic locus notch homolog protein 1) has CLIFI values largely centred around 0 except for brain lower grade gliomas, however while it may appear its expression may not relate to differences between other cancers, its distributions are considerably different from the MTOR (mammalian target of rapamycin) protein which is not associated with any class separation in any model.

**Fig. 5:**
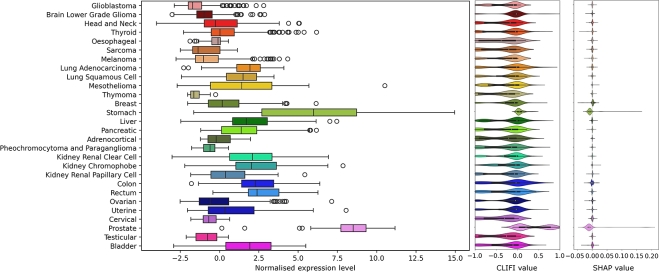
Comparison of protein expression level for MYH11, CLIFI value from LAVASET model, and SHAP value (from TreeExplainer).

**Fig. B.8:**
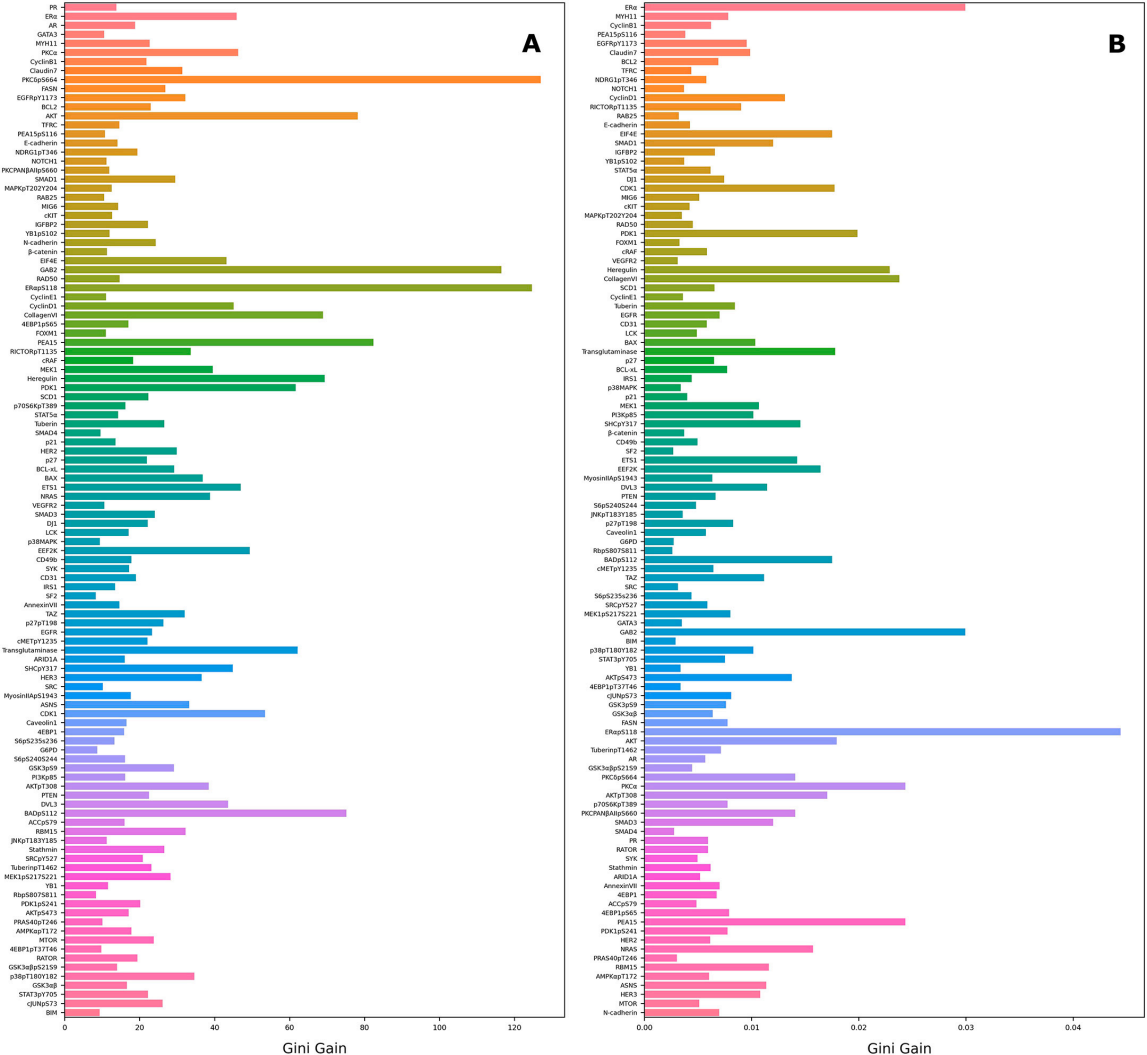
Bar plots of the Gini coefficients of the models with highest performance. Proteins ordered based on the absolute CLIFI values of the model. (A) Random Forest. (B) LAVASET.

**Fig. C.11:**
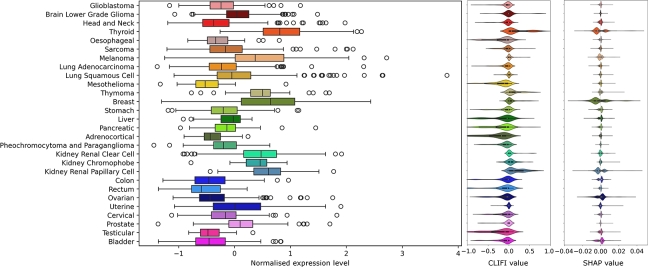
Comparison of protein expression level for BCL2, CLIFI value from LAVASET model, and SHAP value (from TreeExplainer).

**Fig. C.12:**
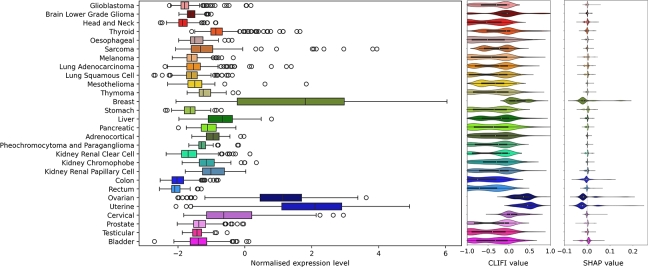
Comparison of protein expression level for ER*α*, CLIFI value from LAVASET model, and SHAP value (from TreeExplainer).

**Fig. C.13:**
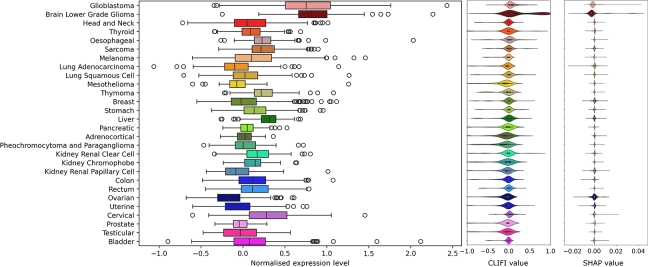
Comparison of protein expression level for NOTCH1, CLIFI value from LAVASET model, and SHAP value (from TreeExplainer).

**Fig. C.14:**
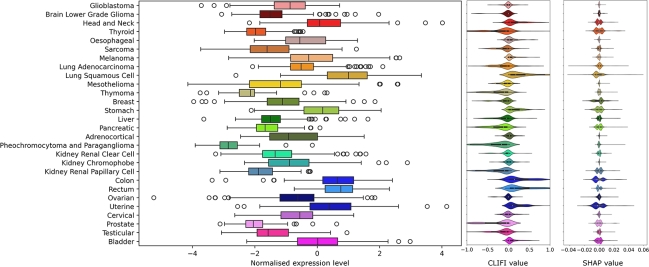
Comparison of protein expression level for TFRC, CLIFI value from LAVASET model, and SHAP value (from TreeExplainer).

**Fig. C.15:**
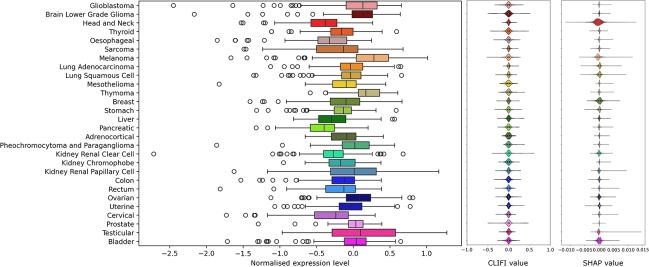
Comparison of protein expression level for MTOR, CLIFI value from LAVASET model, and SHAP value (from TreeExplainer).

We have observed that the CLIFI values relate to the protein expression levels in the original data. For example, for MYH11 (Fig. 5) prostate and stomach cancer have the highest CLIFI values matching the protein expression levels. Likewise, oesophageal, thymoma, cervical, and testicular cancers have CLIFI values indicating lower expression levels. Though mesothelioma, liver, prostate and some other cancer types exhibit bimodal (or multimodal) distributions pointing towards possible heterogeneity in the proteomic signature of these cancers. SHAP values are given for comparison. The same comparison is given in Fig. C.11, Fig. C.12, Fig. C.13, Fig. C.14, Fig. C.15 for the same proteins as shown in Fig. 4. The Gini values, that unlike CLIFI and SHAP do not show directionality, are given for comparison for the top performing models in Fig. B.8.

#### 3.2.5 TCGA data - visualisation of heterogenity in classification of cancer types

**Fig. 6:**
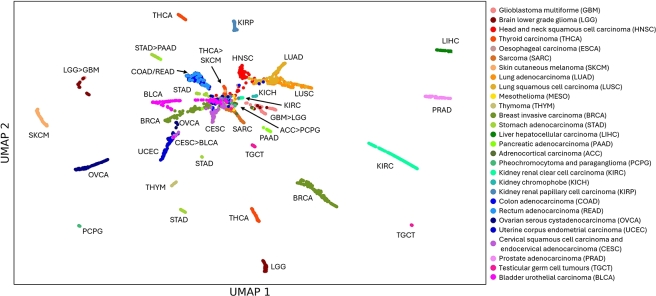
UMAP visualisation of the LAVASET proximity matrix with each cancer type labelled showing heterogeneity of some cancer types in the model prediction. *A* > *B* indicates cluster contains more samples from cancer type A than cancer B, ‘/’ indicates completely overlapped samples within a cluster.

The model performance is given with performance values for each cancer types, however, this fails to show the homo- and heterogeneity of the data. We calculate a proximity matrix from the output from the ensemble model which represents the frequency that pairs of samples end up in the same leaf node in the classification model. We graphically represent the proximities as input to UMAP (2 dimensions) in Fig. 6. The CLIFI values for several proteins indicated that breast cancer display bimodal distributions, and the UMAP plot shows that there appear to be multiple breast and stomach cancer subgroups based on the proteomics-based proximity matrix from LAVASET and visualises the similarity between cancer types based on the model predictions.

While ovarian and uterine cancer had similar CLIFI values to breast cancer for some proteins (e.g. ER*α*), overall, they cluster separately from breast cancer. The UMAP visualisation also shows that colon and rectal cancer (that are indistinguishable) are similar to two clusters of stomach cancer samples, the two lung cancers are closely related to head and neck cancers, and there are 2 clusters of brain cancers (one with majority of glioblastoma samples, another with majority of lower grade gliomas), all of which relates to the relative proximity of these cancers in the body.

## 4 Discussion

We have introduced a new directional feature importance metric, CLIFI, that is integrated within the algorithm. The distribution of CLIFI values contains information on class separation and can be represented both on a global and class-specific level. Therefore, combined with being embedded within the training procedure, it provides unique insights that other methods such as the Gini coefficient and SHAP values do not. More specifically, it allows the identification of heterogeneity within a class for specific features. Secondly, we have extended the LAVA framework to gradient boosting methods and introduced the LAVABOOST algorithm here for datasets with correlated features and multiple classes. While our results indicate that for these datasets RF and LAVASET outperform GBDTs and LAVABOOST, all methods presented in this study outperform other previously published methods, for some cancer types in the TCGA proteomics data.

The CLIFI calculation is based on measuring the change in class distribution of the training dataset during tree building, i.e. a model explainer. Therefore, it is not dependent on the class prediction accuracy and provides a new level of interpretability of stochastic ensemble models. Unlike the Gini coefficient, also arising from the training dataset, we demonstrated that CLIFI values are (significantly) higher for real features than random ones for the Iris dataset. Additionally, when features contain little class information (as seen with the Iris-permuted dataset) the CLIFI values are tightly clustered around 0, while the Gini coefficients remain large. The same was shown for the TCGA data where unimportant features have narrow CLIFI distributions around 0 (e.g. MTOR) and predictive features such as BCL2, ER*α*, NOTCH1, and MYH11 deviate from this. Although, this may suggest that CLIFI is correlated with accuracy, while both RF and LAVASET assign a strikingly high importance to ER*α* for classifying rectum adenocarcinoma, their overall F1-scores of 0 and 4.65% for RF and LAVASET, respectively, indicate this is unrelated. Hence, CLIFI is unaffected by unbalanced classes, unlike other commonly used methods such as Gini and SHAP values, as it treats the dataset as a set of classes rather than one population, making it ideally suited as a model explainer opposed to a prediction explainer. In contrast, Gini can be biased towards features relevant to the dominant class(es).

Given the shift of cancer medical practice towards the need of identifying subpopulations within cancer types [7], [8], the CLIFI distributions lend themselves more appropriate to this type of evaluation for individual classes. We have shown here that it can be used to identify features with bi- or multimodal distributions within a class, even though overall the class could appear as homogeneous. Overall, CLIFI appears to be non-inferior to SHAP in terms of identifying important features, while having the benefit of producing values that are directionally congruent with the patterns observed in the input data (here TCGA protein expression), allowing it to effectively explain the ensemble model's decision making process. We also demonstrated that ablating the least important features (low absolute CLIFI) improves the F1-Score (+0.34%), suggesting that CLIFI can also assist in guiding the process of recursive feature elimination [31] for ensemble models.

While our aim here was to demonstrate the new methodology, the use of the publicly available TCGA data [26] allow comparison of model outputs with previously published literature. For example, for uterine corpus endometrial carcinoma LAVASET [25] outperforms the other methods, and the proteins with the highest magnitude of average naCLIFI values (MYH11, ER*α*, and cyclin B1) have all been found to be expressed in the same direction as those identified by others in endometrial cancers [32], [33], [34].

The original LAVASET method was applied on different datasets (^1^H NMR (1D vector), ECG (8 cyclical leads), CMR imaging (3D mesh)), but with distances defined by the data itself (spatial or temporal) [25]. Here we integrated protein interaction data into this step to allow the method to make the decisions based on topological (pathway) information. Analysis of the top proteins with the most interactions identifies pathways which are also important for disease progression in cancer, such as epidermal growth factor signalling (via EGFR and EGFRpY1173) [35], the MAPK pathway (via p38MAPK and MAPKpT202Y204) [36], and the BCL-regulated apoptotic pathway (via BCL-2 and BCL-xL) [37]. However, not all proteins associated with each pathway are identified as being important. Since we used a multi-class model, the feature importance profile will highlight the most differentiating proteins, and thus will not be an exact match to proteomic profiles identified in biological studies. Additionally, proteins can be part of multiple pathways, hence this impacts the ‘neighbours’ used to calculate the latent feature embedding in ‘LAVA’.

While the UMAP visualisation of model proximities has shown some heterogeneity and subclasses within some cancer types (e.g. breast and stomach cancer), analysis of the CLIFI values allows to identity the features responsible for this (bimodal distributions of ER*α* and others). However, despite MYH11 being the most important protein for prostate cancer prediction and the CLIFI values indicating a bimodal distribution, the UMAP shows only one cluster. Differential ER*α* expression has previously been reported in prostate cancer patients [38], whereas, to the best of our knowledge, differential MYH11 expression has not;the results shown here would therefore warrant further investigation. The CLIFI distribution also allows for further *a posteriori* analysis, such as tests for normality, deviation from 0, and others using conventional, univariate statistical tests. When comparing CLIFI values between models, as done here, the values should be normalised to allow for direct comparison. Whereas for the more common case where only a single model is calculated, the aggregated CLIFI values can be used as is.

Finally, to conclude our analysis, we also extended the ‘LAVA’ framework to boosting methods. For the TCGA data, we observed that LAVASET outperformed LAVABOOST in the prediction of most (but not all) cancer classes. Overall, RF and LAVASET appear to be the most predictive models for this dataset, hence their CLIFI values are potentially of higher interest. However, we also showed that no model outperformed all other models for all classes (and previously showed that LAVASET is non-inferior to RF [25]). Therefore, there may be other datasets for which boosting algorithms do outperform the bagging ones, but demonstrating this was not part of our aims. CLIFI can be implemented in other methods such as XGBoost [39] which may have higher performance. For our data, RF and LAVASET had the highest performance with F1-scores of 92.63% and 92.05%, respectively. However, optimising the additional parameters of XGBoost (same random state) had an F1 of 92.53% showing the improvement over GBDTs for these data (F1 = 85.65%). Since the LAVABOOST outperformed GBDT, it is plausible that the performance of XGBoost may increase further with the addition of the ‘LAVA’ step and the integration of CLIFI will improve its interpretability. Another potential extension would be to explain the predictions of external data using CLIFI (akin to how SHAP aims to explain the predictions), the calculation for CLIFI would be the same for a test set except that in this case the ancestor set is the test set distributions. This remains to be investigated as part of future work; one possible issue would be that it is not applicable to single-sample predictions, as SHAP can be, but it can extend CLIFI as a prediction explainer.

In summary, we have contributed an integrated, directional feature importance metric for decision tree-based models to facilitate feature importance assessment for multi-class classification (‘model explainer’). This metric can be used together with incorporating topological information into the decision functions of tree-based algorithms. The incorporation of topological information has been extended here to protein interactions but can be used with any type of network information that links together individual features to add inductive bias into the model for improved interpretability. Finally, several findings from analysing the individual directional feature importances match with existing literature, while other observations do not, generating potentially new testable hypotheses for cancer heterogeneity and subsequent clinical diagnostic studies.

## Funding

M.K. is supported by the 10.13039/501100000761Imperial College London funded RIPEN Hub project (reference number PSR661). J.M.P. is supported by the Horizon Europe CoDiet project (101084642) and 10.13039/501100000265Medical Research Council (MRC) funded GI-tools project (MR/V012452/1). The CoDiet project is funded by the 10.13039/501100000780European Union under 10.13039/100018693Horizon Europe grant number 101084642. CoDiet research activities taking place at Imperial College London and the University of Nottingham are supported by 10.13039/100014013UK Research and Innovation (UKRI) under the UK government's 10.13039/100018693Horizon Europe funding guarantee (grant number 101084642).

## CRediT authorship contribution statement

**Eloisa Rocha Liedl:** Writing – original draft, Visualization, Validation, Software, Investigation, Formal analysis, Data curation. **Shabeer Mohamed Yassin:** Investigation, Formal analysis, Data curation. **Melpomeni Kasapi:** Writing – review & editing, Supervision, Software, Project administration, Methodology, Conceptualization. **Joram M. Posma:** Writing – review & editing, Visualization, Validation, Supervision, Resources, Project administration, Methodology, Funding acquisition, Conceptualization.

## Declaration of Competing Interest

The authors declare no conflict of interest. The funders had no role in the design of the study; in the collection, analyses, or interpretation of data; in the writing of the manuscript, or in the decision to publish the results.

## Figures and Tables

**Table A.5:** Classification report of RF and LAVASET when classifying 28 cancer types for a single initialisation. Values in bold indicate the highest performance (expressed as percentage) for each metric per cancer type across the 4 methods (see Table A.6 for results for GBDT and LAVABOOST).

Cancer type	Precision		Recall		F1-score		Support all
	RF	LAVASET	RF	LAVASET	RF	LAVASET	
Glioblastoma	95.77	95.71	93.15	91.78	94.44	93.71	73
Brain lower grade glioma	96.97	96.97	97.71	97.71	97.34	97.34	131
Head and Neck	94.59	89.66	98.13	97.2	96.33	93.27	107
Thyroid	96.64	96.64	100	100	98.29	98.29	115
Esophageal	77.27	75	89.47	86.84	82.93	80.49	38
Sarcoma	90.67	85.71	100	97.06	95.1	91.03	68
Melanoma	95.28	98	95.28	92.45	95.28	95.15	106
Lung adenocarcinoma	84.96	85.71	87.27	87.27	86.1	86.49	110
Lung squamous cell	85.57	83.67	83.84	82.83	84.69	83.25	99
Mesothelioma	100	100	73.68	73.68	84.85	84.85	19
Thymoma	100	100	92.59	96.3	96.15	98.11	27
Breast	95.45	95.12	98.91	98.91	97.15	96.98	276
Stomach	94.64	95.5	98.15	98.15	96.36	96.8	108
Liver	92.86	89.09	92.86	87.5	92.86	88.29	56
Pancreatic	96.77	90.91	83.33	83.33	89.55	86.96	36
Adrenocortical	92.31	100	85.71	85.71	88.89	92.31	14
Pheochromocytoma and Paraganglioma	95.83	100	92	96	93.88	97.96	25
Kidney renal clear cell	98.61	96.6	98.61	98.61	98.61	97.59	144
Kidney chromophobe	100	94.44	94.74	89.47	97.3	91.89	19
Kidney renal papillary cell	90.91	92.42	92.31	93.85	91.6	93.13	65
Colon	71.53	72.14	94.5	92.66	81.42	81.12	109
Rectum	0	33.33	0	2.5	0	4.65	40
Ovarian	99.21	98.4	96.92	94.62	98.05	96.47	130
Uterine endometrial	95.31	94.03	92.42	95.45	93.85	94.74	132
Cervical	97.37	97.3	71.15	69.23	82.22	80.9	52
Prostate	100	100	100	100	100	100	106
Testicular germ cell	94.87	97.3	100	97.3	97.37	97.3	37
Bladder urothelial	93.27	95.1	94.17	94.17	93.72	94.63	103

Weighted accuracy	91.92	**91.97**	**93.01**	92.49	**92.29**	91.83	2345

**Table A.6:** Classification report of GBDT and LAVABOOST when classifying 28 cancer types for a single initialisation. Values in bold indicate the highest performance (expressed as percentage) for each metric per cancer type across the 4 methods (see Table A.5 for results for RF and LAVASET).

Cancer type	Precision		Recall		F1-score		Support all
	GBDT	LAVABOOST	GBDT	LAVABOOST	GBDT	LAVABOOST	
Glioblastoma	93.85	96.97	83.56	87.67	88.41	92.09	73
Brain lower grade glioma	93.48	94.16	98.47	98.47	95.91	96.27	131
Head and Neck	86.79	85.09	85.98	90.65	86.38	87.78	107
Thyroid	92.74	96.64	100	100	96.23	98.29	115
Esophageal	75.76	76.67	65.79	60.53	70.42	67.65	38
Sarcoma	73.97	85.92	79.41	89.71	76.6	87.77	68
Melanoma	92.08	98.94	87.74	87.74	89.86	93	106
Lung adenocarcinoma	83.18	82.2	80.91	88.18	82.03	85.09	110
Lung squamous cell	78.64	83.16	81.82	79.8	80.2	81.44	99
Mesothelioma	85.71	100	31.58	15.79	46.15	27.27	19
Thymoma	100	100	85.19	88.89	92	94.12	27
Breast	79.13	78	98.91	98.91	87.92	87.22	276
Stomach	90.18	98.15	93.52	98.15	91.82	98.15	108
Liver	88.68	86.21	83.93	89.29	86.24	87.72	56
Pancreatic	96.3	93.75	72.22	83.33	82.54	88.24	36
Adrenocortical	100	100	71.43	85.71	83.33	92.31	14
Pheochromocytoma and Paraganglioma	96.15	96	100	96	98.04	96	25
Kidney renal clear cell	88.12	95.95	97.92	98.61	92.76	97.26	144
Kidney chromophobe	100	100	89.47	89.47	94.44	94.44	19
Kidney renal papillary cell	93.65	96.67	90.77	89.23	92.19	92.8	65
Colon	70.07	70.14	88.07	92.66	78.05	79.84	109
Rectum	0	0	0	0	0	0	40
Ovarian	90.84	95.2	91.54	91.54	91.19	93.33	130
Uterine endometrial	86.51	92.19	82.58	89.39	84.5	90.77	132
Cervical	87.88	77.5	55.77	59.62	68.24	67.39	52
Prostate	99.05	99.05	98.11	98.11	98.58	98.58	106
Testicular germ cell	97.22	100	94.59	97.3	95.89	98.63	37
Bladder urothelial	85.54	97.65	68.93	80.58	76.34	88.3	103

Weighted accuracy	85.64	88.42	86.57	89.08	85.6	88.15	2345

**Fig. C.10:**
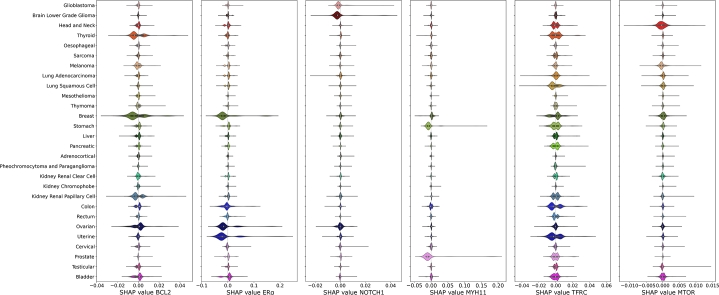
Visualisation of SHAP values with violin plots of five selected top proteins, and one unimportant, same as shown for CLIFI from the LAVASET multi-class classification model in Fig. 4. From left to right: BCL2, ER*α*, NOTCH1, MYH11, TFRC, MTOR.
